# Mortality by Age, Gender, and Race and Ethnicity in People Experiencing Homelessness in Boston, Massachusetts

**DOI:** 10.1001/jamanetworkopen.2023.31004

**Published:** 2023-08-31

**Authors:** Danielle R. Fine, Kirsten A. Dickins, Logan D. Adams, Nora K. Horick, Natalia Critchley, Katherine Hart, Jessie M. Gaeta, Elizabeth Lewis, Sara E. Looby, Travis P. Baggett

**Affiliations:** 1 Division of General Internal Medicine, Department of Medicine, Massachusetts General Hospital, Boston; 2 Harvard Medical School, Boston, Massachusetts; 3 Community, Systems and Mental Health Nursing Department, Rush University Medical Center, Chicago, Illinois; 4 Yvonne L. Munn Center for Nursing Research, Massachusetts General Hospital, Boston; 5 Biostatistics Center, Massachusetts General Hospital, Boston; 6 The Institute for Research, Quality, and Policy in Homeless Health Care, Boston Health Care for the Homeless Program, Boston, Massachusetts; 7 Boston University School of Medicine, Boston, Massachusetts; 8 Boston University School of Public Health, Boston, Massachusetts; 9 Metabolism Unit, Endocrinology Division, Department of Medicine, Massachusetts General Hospital, Boston

## Abstract

**Importance:**

People experiencing homelessness (PEH) face disproportionately high mortality rates compared with the general population, but few studies have examined mortality in this population by age, gender, and race and ethnicity.

**Objective:**

To evaluate all-cause and cause-specific mortality in a large cohort of PEH by age, gender, and race and ethnicity.

**Design, Setting, and Participants:**

An observational cohort study was conducted from January 1, 2003, to December 31, 2018. All analyses were performed between March 16, 2021, and May 12, 2022. A cohort of adults (age ≥18 years) seen at the Boston Health Care for the Homeless Program (BHCHP), a large federally funded Health Care for the Homeless organization in Boston, Massachusetts, from January 1, 2003, to December 31, 2017, was linked to Massachusetts death occurrence files spanning January 1, 2003, to December 31, 2018.

**Main Outcomes and Measures:**

Age-, gender-, and race and ethnicity–stratified all-cause and cause-specific mortality rates were examined and compared with rates in the urban Northeast US population using mortality rate ratios (RRs).

**Results:**

Among the 60 092 adults included in the cohort with a median follow-up of 8.6 (IQR, 5.1-12.5) years, 7130 deaths occurred. The mean (SD) age at death was 53.7 (13.1) years; 77.5% of decedents were men, 21.0% Black, 10.0% Hispanic/Latinx, and 61.5% White. The all-cause mortality rate was 1639.7 deaths per 100 000 person-years among men and 830 deaths per 100 000 person-years among women. The all-cause mortality rate was highest among White men aged 65 to 79 years (4245.4 deaths per 100 000 person-years). Drug overdose was a leading cause of death across age, gender, and race and ethnicity groups, while suicide uniquely affected young PEH and HIV infection and homicide uniquely affected Black and Hispanic/Latinx PEH.

**Conclusions and Relevance:**

In this large cohort study of PEH, all-cause and cause-specific mortality varied by age, gender, and race and ethnicity. Tailored interventions focusing on those at elevated risk for certain causes of death are essential for reducing mortality disparities across homeless-experienced groups.

## Key Points

**Question:**

What are the mortality rates and causes of death by age, gender, and race and ethnicity in a large cohort of people experiencing homelessness (PEH)?

**Findings:**

In this cohort study of 60 092 PEH, all-cause mortality rates differed by age, gender, and race and ethnicity. Drug overdose was a leading cause of death across age, gender, and race and ethnicity groups, while suicide uniquely affected young PEH and HIV infection and homicide uniquely affected Black and Hispanic/Latinx PEH.

**Meaning:**

The findings of this study suggest that interventions to reduce mortality disparities among PEH should consider the unique health needs across sociodemographic groups.

## Teaser

This cohort study examines all-cause and cause-specific mortality in adults experiencing homelessness by age, gender, and race and ethnicity.

## Introduction

Homelessness in the US has been growing over the past several years. Between 2019 and 2020, the number of people experiencing homelessness (PEH) increased by 2% with an estimated 580 445 individuals experiencing homelessness on a single night in 2020.^1^ The number of PEH in the US has continued to increase during the COVID-19 pandemic^2^ and is projected to continue increasing amid a national eviction and housing crisis.^3^

People experiencing homelessness face a disproportionate burden of complex medical and psychosocial challenges,^4,5^ resulting in severe morbidity and premature mortality. Prior studies conducted in various US cities have documented death rate disparities, with PEH dying at significantly higher rates and at earlier ages than the general population.^6,7,8,9,10,11,12,13^ The biopsychosocial complexity of homelessness is underscored by the nature and magnitude of the leading causes of death observed in this population. A 1988-1993 mortality study of homeless-experienced individuals in Massachusetts documented the toll of HIV/AIDS deaths in this population.^8^ In a 2003-2008 study in the same setting, drug overdose surpassed HIV/AIDS as the leading cause of death. Drug overdose mortality rates have since far exceeded the general population,^6,14^ illustrating the extent to which PEH bear a disproportionate burden of public health crises.

Although overall mortality disparities with the general population have been documented, studies assessing mortality by demographic group are limited.^15,16^ In a 1991-2001 mortality study of the Canadian population, mortality was substantially higher among people living in shelters, rooming houses, and hotels, particularly among men.^15^ A 2000-2009 study evaluating mortality among unsheltered homeless adults in Boston, Massachusetts, found higher rates of death among men compared with women and among non-Hispanic White individuals compared with non-Hispanic Black individuals.^16^

To help inform important equity-focused policy decisions, we developed one of the largest mortality databases of PEH to date. In a recently published study using this database, members of our study team described temporal mortality trends, reporting a widening mortality gap between PEH and the general population over time.^13^ The objective of this current analysis was to evaluate all-cause and cause-specific mortality rates by age, gender, and race and ethnicity, and compare these rates with those in the general US population. A comprehensive assessment of mortality among demographic subgroups of PEH could inform important policy efforts addressing long-standing mortality disparities in this diverse population.

## Methods

### Study Population

The MassGeneral Brigham Human Research Committee Institutional Review Board approved this study with a waiver of informed consent due to the low-risk nature of the study. As described previously, we assembled a cohort of individuals aged 18 years or older who had an in-person encounter at Boston Health Care for the Homeless Program (BHCHP) between January 1, 2003, and December 31, 2017.^13,14^ The BHCHP is a federally qualified health center in Boston, Massachusetts, that serves more than 11 000 PEH annually in more than 140 000 medical, behavioral, and oral health encounters.^17^ The mission of BHCHP is to “ensure unconditionally equitable and dignified access to the highest quality health care for all individuals and families experiencing homelessness.”^18^ Although individuals are homeless when they enroll in care at BHCHP, many patients transition into and out of homelessness over time. Therefore, the BHCHP cohort consisted of homeless-experienced individuals. We extracted sociodemographic information from the BHCHP electronic health record, including age, gender, and race and ethnicity. Gender and race and ethnicity were based on self-report at the time of initial BHCHP enrollment. Race and ethnicity was included in the analysis to examine how mortality may differ by race and ethnicity. Race and ethnicity were categorized as American Indian/Alaska Native; Asian/Pacific Islander; Black, non-Hispanic/Latinx; Hispanic/Latinx; White, non-Hispanic/Latinx; more than 1 race; and unknown. Nine percent of the cohort had missing race and ethnicity data. These individuals were included in the full cohort estimates but were excluded from the race and ethnicity–stratified estimates. We excluded individuals who were older than 79 years at the time of death from our analyses due to the limited number of people within this age group. We followed the Strengthening the Reporting of Observational Studies in Epidemiology (STROBE) reporting guideline for this observational study.

### Ascertainment of Death Occurrences and Causes of Death

We used Match*Pro, version 1.6.3 (National Cancer Institute) to link the BHCHP cohort with the Massachusetts Department of Public Health (MDPH) Registry of Vital Records and Statistics (RVRS) death occurrence files. Death occurrence files spanned from January 1, 2003, to December 31, 2018, to allow for at least 1 year of follow-up past the final study entry date. Our linkage procedure used first name, last name, date of birth, and social security number. Two of us (D.R.F. and K.A.D.) independently conducted a manual review of each pair identified by Match*Pro between the BHCHP cohort and MDPH RVRS death occurrence files that achieved a threshold score greater than or equal to 0.65^19^ (κ>0.99). The investigators accepted pairs as a true match if there was alignment on 1 or more of the following National Death Index criteria^20^: (1) social security number; (2) first name, last name, and month and year of birth (±1 year); and (3) first name, last name, and month and day of birth. A third investigator (T.P.B.) adjudicated discrepancies. These methods have been used in prior studies investigating mortality in homeless-experienced cohorts.^6,16,21,22^

Cause-specific mortality analyses were based on underlying cause of death, which is listed as an *International Classification of Diseases and Related Health Problems, Tenth Revision* (*ICD-10*) code in the MDPH RVRS death occurrence files.^23,24^ Investigators grouped *ICD-10* codes into clinically relevant cause-of-death categories according to the Centers for Disease Control and Prevention classification system (eTable 1 in Supplement 1).^24^ There were 4 individuals with a missing underlying cause of death. We used the urban Northeast US adult (aged 18-79 years) population as the comparator reference group. We obtained 2003-2018 mortality data on this reference population from the Centers for Disease Control and Prevention Wide Ranging Online Data for Epidemiologic Research files.^25^

### Statistical Analysis

All analyses were performed between March 16, 2021, and May 12, 2022. We calculated age-, gender-, and race and ethnicity–stratified all-cause mortality rates aggregated across study years, reported as the number of deaths per 100 000 person-years with 95% CIs based on the normal distribution. We compared these results with the reference population by calculating mortality rate ratios (RRs) (mortality rate in the BHCHP cohort divided by the mortality rate in the reference population) with 95% CIs.^26^ We repeated this process for the 5 leading causes of death in each demographic subgroup. We also calculated mortality rate differences (mortality rate in the BHCHP cohort minus the mortality rate in the reference population) with 95% CIs (eTable 2 and eTable 3 in Supplement 1). We do not report leading causes of death for which the count was less than 3 due to patient confidentiality and low precision of estimates when calculating RRs. We do not report estimates for American Indian/Alaskan Natives, Asian/Pacific Islanders, and individuals who identified as more than 1 race because of the limited number of deaths within each of these categories. We conducted all analyses with SAS, version 9.4 (SAS Institute Inc). We used a 2-sided significance level of *P* < .05.

## Results

A total of 60 092 patients were seen at BHCHP during the study period with a median of 8.6 (IQR, 5.1-12.5) years of follow-up from study entry to death or study exit, yielding 520 430 person-years of follow-up.

Characteristics of the entire BHCHP cohort are reported in Table 1. The mean (SD) age at study entry was 40.4 (13.1) years. Among decedents, the mean age at death was 53.7 (13.1) years, 77.5% were men, 22.5% were women, 21.0% were non-Hispanic/Latinx Black, 10.0% Hispanic/Latinx, and 61.5% non-Hispanic/Latinx White. Among decedents in the reference population, 57.0% were men, 29.8% were non-Hispanic/Latinx Black, 13% were Hispanic/Latinx, and 52.0% were non-Hispanic/Latinx White.

**Table 1.** Characteristics of Adults Seen at Boston Health Care for the Homeless Program Between 2003 and 2017

Characteristic	Entire BHCHP cohort (n = 60 092)	Nondeceased (n = 52 962)	Deceased (n = 7130)
Age at cohort entry, mean (SD), y	40.4 (13.1)	39.4 (12.8)	47.9 (12.5)
Gender, No. (%)			
Men	38 084 (63.4)	32 555 (61.5)	5529 (77.5)
Women	22 008 (36.6)	20 407 (38.5)	1601 (22.5)
Race and ethnicity, No. (%)			
American Indian/Alaska Native, non-Hispanic/Latinx	316 (0.5)	283 (0.5)	33 (0.5)
Asian/Pacific Islander, non-Hispanic/Latinx	749 (1.2)	708 (1.3)	41 (0.6)
Black, non-Hispanic/Latinx	15 928 (26.5)	14 428 (27.2)	1500 (21.0)
Hispanic/Latinx	10 773 (17.9)	10 063 (19.0)	710 (10.0)
White, non-Hispanic/Latinx	26 364 (43.9)	21 978 (41.5)	4386 (61.5)
More than 1 race, non-Hispanic/Latinx	484 (0.8)	456 (0.9)	28 (0.4)
Unknown race, non-Hispanic/Latinx	5478 (9.1)	5046 (9.5)	432 (6.1)
Age at death, mean (SD), y	NA	NA	53.7 (13.1)
Autopsy performed, No. (%)			
Yes/partial	NA	NA	2416 (33.9)
No	NA	NA	4516 (63.3)
Unknown/not classifiable	NA	NA	198 (2.8)
Place of death, No. (%)	NA	NA	
Hospital	NA	NA	3016 (42.3)
Nursing home/assisted living	NA	NA	1897 (26.6)
Residence	NA	NA	908 (12.7)
Hospice	NA	NA	547 (7.7)
Dead on arrival to hospital	NA	NA	357 (5.0)
Other	NA	NA	399 (5.6)
Unknown	NA	NA	6 (0.1)

### All-Cause Mortality by Age, Gender, and Race and Ethnicity

**Figure 1.**
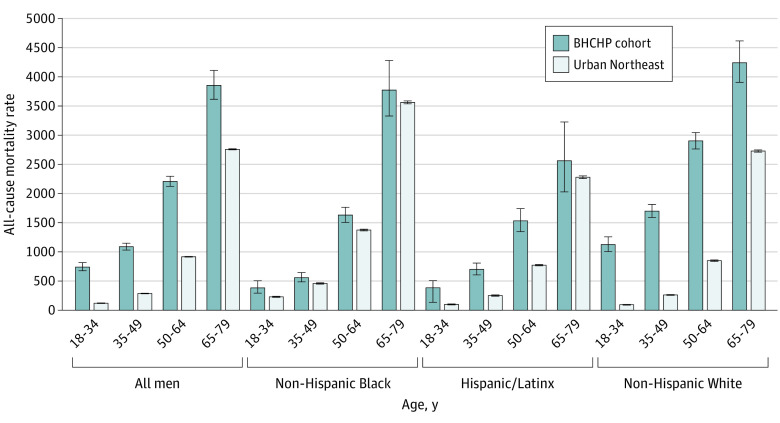
All-Cause Mortality Rates Among Men in the Boston Health Care for the Homeless Program Cohort Compared With Men in the Urban Northeast US Population, 2003-2018 Urban Northeast reference population data obtained from the Centers for Disease Control and Prevention Wide Ranging Online Data for Epidemiologic Research files. Error bars indicate 95% CI.

**Figure 2.**
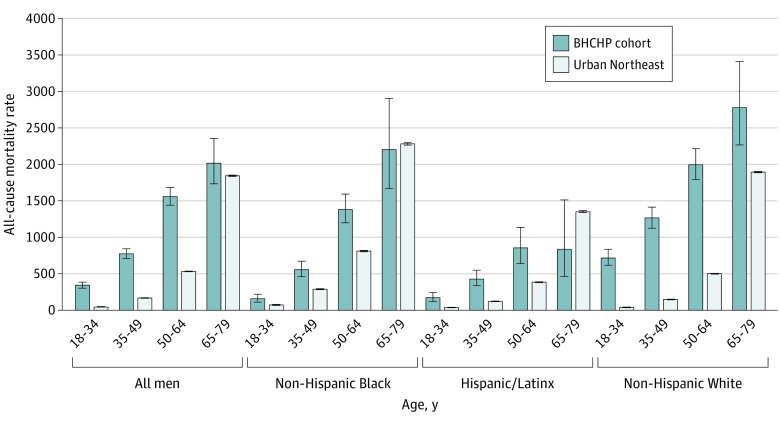
All-Cause Mortality Rates Among Women in the Boston Health Care for the Homeless Program Cohort Compared With Women in the Urban Northeast US Population, 2003-2018 Urban Northeast reference population data obtained from the Centers for Disease Control and Prevention Wide Ranging Online Data for Epidemiologic Research files. Error bars indicate 95% CI.

Aggregated across all study years, the all-cause mortality rate was 1639.7 per 100 000 person-years among men and 830 per 100 000 person years among women. White men aged 65 to 79 years experienced the largest all-cause mortality rate (4245.4 deaths per 100 000 person-years) (Figure 1). Compared with the general population, the largest all-cause mortality RR was among White women aged 18 to 34 years (RR, 18.4; 95% CI, 15.8-21.5) (Figure 2). All-cause mortality rates increased with age, while all-cause mortality RRs decreased with age. All-cause mortality RRs were larger among women than men and were higher among White individuals than Black and Hispanic individuals.

### Cause-Specific Mortality by Age, Gender, and Race and Ethnicity

Drug overdose was the leading cause of death among BHCHP men and women younger than 50 years across races and ethnicities (Table 2 and Table 3). The drug overdose mortality rate was highest among White men aged 18 to 34 years (797.7 per 100 000 person-years), while the largest drug overdose mortality RR was among White women aged 18 to 34 years (RR, 39.7; 95% CI, 32.6-48.3) (Table 3). Cancer and cardiovascular disease were among the leading causes of death in nearly every demographic group aged 50 years or older (Table 2 and Table 3).

**Table 2.** Mortality Rates for the 5 Leading Causes of Death Among Men in the BHCHP Cohort Compared With Men in the Urban Northeast US Population, 2003-2018^a^

COD rank by age strata	Entire BHCHP cohort			BHCHP cohort stratified by race and ethnicity								
	COD	No. (crude rate)	RR (95% CI)	Black, non-Hispanic/Latinx			Hispanic/Latinx			White, non-Hispanic/Latinx		
				COD	No. (crude rate)	RR (95% CI)	COD	No. (crude rate)	RR (95% CI)	COD	No. (crude rate)	RR (95% CI)
**Age strata, 18-34 y**												
First	Drug overdose	291 (487.6)	23.0 (20.5-25.9)	Drug overdose	15 (113.1)	11.4 (6.8-18.9)	Drug overdose	29 (219.6)	14.4 (10.0-20.8)	Drug overdose	213 (797.7)	23.6 (20.6-27.0)
Second	Suicide	29 (48.6)	4.1 (2.9-5.9)	Homicide	12 (90.4)	0.8 (0.5-1.4)	PSUD	6 (45.4)	20.8 (9.2-46.8)	Suicide	22 (82.4)	6.0 (3.9-9.1)
Third	PSUD	29 (48.6)	36.0 (24.8-52.4)	CVD	5 (37.7)	2.6 (1.1-6.2)	Ill-defined	3 (22.7)	7.6 (2.4-23.7)	PSUD	19 (71.2)	54.8 (34.2-87.8)
Fourth	Homicide	21 (35.2)	1.1 (0.7-1.7)	Suicide	3 (22.6)	1.8 (0.6-5.4)	Homicide	3 (22.7)	1.0 (0.3-3.2)	Ill-defined	15 (56.2)	14.7 (8.8-24.6)
Fifth	Ill-defined	19 (31.8)	8.7 (5.5-13.7)	Cancer	3 (22.6)	2.6 (0.8-8.0)	NA	NA	NA	Transport accident	7 (26.2)	2.6 (1.2-5.4)
**Age strata, 35-49 y**												
First	Drug overdose	544 (436.7)	11.3 (10.4-12.3)	Drug overdose	49 (144.9)	4.0 (3.0-5.3)	Drug overdose	84 (317.0)	8.7 (7.0-10.8)	Drug overdose	364 (703.6)	14.9 (13.4-16.5)
Second	CVD	145 (116.4)	2.0 (1.7-2.4)	CVD	34 (100.6)	1.0 (0.7-1.3)	CVD	17 (64.2)	1.6 (1.0-2.6)	CVD	85 (164.3)	3.3 (2.6-4.0)
Third	PSUD	102 (81.9)	9.6 (7.9-11.7)	Cancer	17 (50.3)	0.9 (0.5-1.4)	PSUD	14 (52.8)	5.1 (3.0-8.6)	PSUD	77 (148.8)	18.0 (14.3-22.7)
Fourth	Cancer	85 (68.2)	1.6 (1.3-2.0)	HIV	14 (41.4)	0.8 (0.5-1.4)	HIV	13 (49.1)	1.9 (1.1-3.3)	Liver disease	55 (106.3)	8.2 (6.3-10.7)
Fifth	Liver disease	73 (58.6)	5.0 (3.9-6.3)	Homicide	11 (32.5)	0.8 (0.5-1.5)	Cancer	10 (37.7)	1.2 (0.6-2.2)	Cancer	48 (92.8)	2.3 (1.7-3.0)
**Age strata, 50-64 y**												
First	CVD	524 (437.4)	1.7 (1.5-1.8)	Cancer	139 (373.4)	1.1 (0.9-1.3)	Drug overdose	43 (280.0)	8.2 (6.1-11.2)	CVD	348 (613.0)	2.5 (2.3-2.8)
Second	Cancer	485 (404.8)	1.6 (1.4-1.7)	CVD	121 (325.1)	0.8 (0.7-1.0)	Cancer	38 (247.4)	1.3 (0.9-1.8)	Cancer	288 (507.3)	1.9 (1.7-2.2)
Third	Drug overdose	388 (323.8)	9.2 (8.3-10.2)	Drug overdose	70 (188.0)	3.3 (2.6-4.1)	Liver disease	26 (169.3)	3.6 (2.5-5.4)	Drug overdose	252 (443.9)	14.1 (12.4-16.0)
Fourth	PSUD	211 (176.1)	10.3 (8.9-11.8)	PSUD	35 (94.0)	3.7 (2.7-5.2)	CVD	25 (162.8)	0.8 (0.6-1.2)	PSUD	156 (274.8)	19.0 (16.1-22.4)
Fifth	Liver disease	201 (167.8)	4.4 (3.8-5.1)	Liver disease	31 (83.3)	2.1 (1.5-3.0)	HIV	14 (91.1)	2.3 (1.3-3.8)	Liver disease	136 (239.6)	6.2 (5.3-7.4)
**Age strata, 65-79 y**												
First	Cancer	260 (1067.1)	1.2 (1.1-1.4)	Cancer	87 (1356.1)	1.3 (1.0-1.6)	Cancer	17 (604.5)	0.9 (0.6-1.5)	CVD	159 (1231.8)	1.4 (1.2-1.7)
Second	CVD	260 (1067.1)	1.2 (1.1-1.3)	CVD	65 (1013.2)	0.8 (0.7-1.1)	CVD	14 (497.8)	0.7 (0.4-1.1)	Cancer	139 (1076.9)	1.2 (1.0-1.4)
Third	Lower resp.	56 (229.8)	1.8 (1.4-2.4)	Diabetes	16 (249.4)	1.5 (0.9-2.4)	Lower resp.	7 (248.9)	3.3 (1.6-7.0)	Lower resp.	36 (278.9)	1.9 (1.4-2.7)
Fourth	Diabetes	39 (160.1)	1.6 (1.2-2.2)	Lower resp.	9 (140.3)	1.1 (0.6-2.1)	PSUD	4 (142.2)	10.1 (3.8-27.3)	Drug overdose	20 (154.9)	30.9 (19.7-48.6)
Fifth	Drug overdose	30 (123.1)	15.3 (10.7-22.1)	Kidney failure^b^	7 (109.1)	1.3 (0.6-2.7)	Cerebrovascular disease	4 (142.2)	1.6 (0.6-4.3)	Other resp. diseases^c^	18 (139.4)	2.5 (1.6-4.0)

Abbreviations: BHCHP, Boston Health Care for the Homeless Program; COD, cause of death; CVD, cardiovascular disease; lower resp., chronic lower respiratory disease; NA, not available; PSUD, psychoactive substance use disorder; RR, rate ratio.

^a^ Psychoactive substance use disorder includes *International Statistical Classification of Diseases and Related Health Problems, 10th Revision* (*ICD-10*) codes for mental and behavioral disorders due to psychoactive substance use (codes F10-19) and is generally intended for deaths related to a chronic pattern or sequelae of substance use rather than acute poisoning, which is documented as drug overdose (codes X40-X44, Y10-Y14).

^b^ Identical number of deaths; cause of death with largest RR displayed. Other cause was cerebrovascular disease, RR, 0.7 (95% CI, 0.3-1.5).

^c^ Other resp. diseases *ICD-10* codes: J00-J06, J20-J22, J30-J39, J60-J70, J80-J84, J85-J86, J90-J94, and J96-J98.

**Table 3.** Mortality Rates for the 5 Leading Causes of Death Among Women in the BHCHP Cohort Compared With Women in the Urban Northeast US Population, 2003-2018^a^

COD rank by age strata	Entire BHCHP cohort			BHCHP cohort stratified by race and ethnicity								
	COD	No. (crude rate)	RR (95% CI)	Black, non-Hispanic/Latinx			Hispanic/Latinx			White, non-Hispanic/Latinx		
				COD	No. (crude rate)	RR (95% CI)	COD	No. (crude rate)	RR (95% CI)	COD	No. (crude rate)	RR (95% CI)
**Age strata, 18-34 y**												
First	Drug overdose	139 (189.7)	27.0 (22.8-32.0)	Drug overdose	6 (29.1)	6.7 (3.0-14.9)	Drug overdose	16 (85.2)	20.8 (12.6-34.4)	Drug overdose	105 (455.9)	39.7 (32.6-48.3)
Second	PSUD	26 (35.5)	59.1 (39.2-88.7)	Diabetes	3 (14.5)	6.7 (3.0-4.9)	PSUD	3 (16.0)	30.1 (9.4-97.0)	PSUD	16 (69.5)	103.7 (61.2-175.5)
Third	Suicide	15 (20.5)	6.1 (3.7-10.2)	Homicide	3 (14.5)	1.8 (0.6-5.6)	NA	NA	NA	Suicide	10 (43.4)	11.0 (5.9-20.6)
Fourth	Ill-defined	9 (12.3)	7.8 (4.0-15.0)	CVD	3 (14.5)	1.9 (0.6-6.1)	NA	NA	NA	Ill-defined	8 (34.7)	21.3 (10.5-43.1)
Fifth	Other accidents^b^	7 (9.6)	1.2 (0.6-2.5)	PSUD	3 (14.5)	19.0 (6.0-60.4)	NA	NA	NA	Other accidents^b^	5 (21.7)	1.8 (0.7-4.3)
**Age strata, 35-49 y**												
First	Drug overdose	199 (300.9)	20.2 (17.5-23.2)	Drug overdose	26 (135.2)	8.0 (5.4-11.7)	Drug overdose	21 (144.8)	16.4 (10.6-25.4)	Drug overdose	140 (586.5)	29.4 (24.8-34.9)
Second	CVD	49 (74.1)	3.1 (2.4-4.1)	CVD	13 (67.6)	1.4 (0.8-2.3)	Liver disease	9 (62.1)	15.5 (8.0-30.2)	PSUD	32 (134.1)	46.3 (32.2-66.5)
Third	PSUD	47 (71.1)	23.7 (17.6-31.7)	Cancer	11 (57.2)	0.8 (0.4-1.4)	CVD	8 (55.2)	3.7 (1.9-7.5)	CVD	25 (104.7)	6.1 (4.1-9.0)
Fourth	Cancer	39 (59.0)	1.2 (0.9-1.6)	HIV	9 (46.8)	1.5 (0.8-2.8)	PSUD	6 (41.4)	15.9 (7.0-35.9)	Cancer	18 (75.4)	1.6 (1.0-2.5)
Fifth	Liver disease	29 (43.8)	8.2 (5.7-11.9)	PSUD^c^	6 (31.2)	7.1 (3.1-15.8)	HIV	5 (34.5)	2.7 (1.1-6.6)	Liver disease	15 (62.8)	9.7 (5.8-16.2)
**Age strata, 50-64 y**												
First	Cancer	115 (280.7)	1.3 (1.1-1.6)	Cancer	47 (343.7)	1.2 (0.9-1.6)	CVD	8 (145.1)	1.8 (0.9-3.7)	Drug overdose	62 (363.5)	26.9 (20.9-34.6)
Second	Drug overdose	98 (239.2)	18.5 (15.1-22.6)	CVD	39 (285.2)	1.4 (1.0-1.9)	Drug overdose	6 (108.8)	13.4 (6.0-29.9)	Cancer	52 (304.8)	1.4 (1.0-1.8)
Third	CVD	97 (236.7)	2.1 (1.7-2.6)	Drug overdose	19 (139.0)	7.3 (4.6-11.4)	Liver disease	6 (108.8)	7.7 (3.4-17.2)	Liver disease	39 (228.6)	14.6 (10.7-20.1)
Fourth	Liver disease	59 (144.0)	9.8 (7.5-12.6)	HIV	10 (73.1)	2.8 (1.5-5.2)	Cancer	5 (90.7)	0.6 (0.3-1.6)	CVD	38 (222.8)	2.5 (1.8-3.4)
Fifth	PSUD	30 (73.2)	17.8 (12.4-25.6)	Liver disease	9 (65.8)	3.8 (2.0-7.4)	PSUD	4 (72.6)	22.5 (8.3-60.7)	Lower resp.	18 (105.5)	5.2 (3.3-8.3)
**Age strata, 65-79 y**												
First	Cancer	50 (611.8)	1.0 (0.7-1.3)	Cancer	18 (792.9)	1.1 (0.7-1.8)	CVD	3 (228.4)	0.6 (0.2-1.7)	Cancer	25 (755.3)	1.1 (0.7-1.6)
Second	CVD	38 (465.0)	0.9 (0.6-1.2)	CVD	12 (528.6)	0.7 (0.4-1.3)	NA	NA	NA	CVD	20 (604.3)	1.2 (0.8-1.9)
Third	Lower resp.	15 (183.6)	1.8 (1.1-3.0)	Cerebrovascular disease	4 (176.2)	1.5 (0.6-4.1)	NA	NA	NA	Lower resp.	12 (362.6)	2.8 (1.6-4.9)
Fourth	Liver disease	8 (97.9)	4.3 (2.2-8.6)	Sepsis	3 (132.2)	2.3 (0.7-7.1)	NA	NA	NA	Liver disease	5 (151.1)	6.4 (2.6-15.3)
Fifth	Cerebrovascular disease	7 (85.7)	1.0 (0.5-2.2)	NA	NA	NA	NA	NA	NA	Viral hepatitis^d^	3 (90.6)	29.8 (9.5-93.1)

Abbreviations: BHCHP, Boston Health Care for the Homeless Program; COD, cause of death; CVD, cardiovascular disease; lower resp., chronic lower respiratory disease; NA, not available; PSUD, psychoactive substance use disorder; RR, rate ratio.

^a^ Psychoactive substance use disorder includes *International Statistical Classification of Diseases and Related Health Problems, 10th Revision* (*ICD-10*) codes for mental and behavioral disorders due to psychoactive substance use (codes F10-19) and is generally intended for deaths related to a chronic pattern or sequelae of substance use rather than acute poisoning, which is documented as drug overdose (codes X40-X44, Y10-Y14).

^b^ Other accidents: *ICD-10* codes W00-X59, Y16-Y36, and Y86.

^c^ Identical number of deaths; cause of death with largest RR displayed. Other cause was lower respiratory disease, RR, 5.3 (95% CI, 2.4-11.9).

^d^ Identical number of deaths; cause of death with largest RR displayed. Other causes were drug overdose, RR, 34.6 (95% CI, 11.1-107.3); diabetes, RR, 1.6 (95% CI, 0.5-5.1); and other digestive diseases, RR, 2.0 (95% CI, 0.7-6.3).

Suicide was a leading cause of death among Black and White men and White women aged 18 to 34 years at rates significantly higher than the general population (RR, 4.1; 95% CI, 2.9-5.9 among men; RR, 6.1; 95% CI, 3.7-10.2 among women) (Table 2 and Table 3). Homicide was a leading cause of death among Black and Hispanic men younger than 50 years at rates that did not differ significantly from the general population (Table 2). HIV infection was a leading cause of death among Black and Hispanic men and women aged 35 to 64 years with RRs significantly higher than in the general population (except among Black individuals aged 35-49 years) (Table 2 and Table 3). Liver disease was a leading cause of death among men aged 35 to 64 years and women aged 35 to 79 years across races and ethnicities. The largest liver disease mortality RR was among Hispanic women aged 35 to 49 years (RR, 15.5; 95% CI, 8.0-30.2) (Table 3).

## Discussion

In this large mortality cohort study of PEH, there were several notable findings. Drug overdose deaths disproportionately affected homeless-experienced individuals across age, gender, and race and ethnicity groups at rates up to 40-fold higher than the general US population. The largest drug overdose mortality disparity occurred in homeless-experienced women aged 18 to 34 years followed by men aged 65 to 79 years, consistent with evidence showing an increasing prevalence of substance use among older homeless adults.^27^ These findings highlight the urgency of destigmatizing substance use, using universal substance use screening, supporting legislative changes that will allow for broader implementation of overdose prevention measures, and deploying innovative treatment delivery models in settings serving PEH.

As mortality is influenced by a complex interplay between biologic, social, behavioral, and environmental factors, it follows that specific causes of death disproportionately impacted certain sociodemographic groups. Suicide was a prevalent cause of death among younger homeless-experienced individuals at rates far exceeding the general population. This finding may support evidence suggesting a relative paucity of protective relationships and social supports among PEH.^28^ Homicide distinctly affected young Black and Hispanic individuals, although the rates were not substantially higher than the general population. This may suggest that the public health crisis of homicide victimization among Black and Hispanic individuals^29^ occurs irrespective of housing status. Black and Hispanic individuals were disproportionately impacted by HIV. This is concordant with HIV-associated mortality in the general population, which may reflect the impact of interpersonal, institutional, and structural racism in HIV prevention, diagnosis, and retention in care.^30^

The differing cause of death profiles in demographic subgroups of the BHCHP cohort suggest that stakeholders should consider the sociodemographic characteristics and contextual experiences of PEH when designing and implementing tailored services with this population.^31,32^ Future investigation should focus on intersectionality,^31,33^ considering the interconnecting social, behavioral, and environmental factors that differentially influence mortality risk in PEH and the potentially mitigating effect of equitable and inclusive housing policies on this risk.

In general, all-cause mortality rates and mortality RRs were highest among White men and women across age strata. These findings are consistent with prior studies reporting larger mortality disparities compared with the general population among White PEH compared with Black and Hispanic PEH^6,7^ and may reflect underlying mechanisms of individual and structural racism. First, the path toward homelessness may differ based on race and ethnicity. Factors such as discriminatory housing policies, unequal economic opportunities, and disproportionate criminal-legal system involvement are more commonly associated with homelessness among people of racial and ethnic groups other than White,^32^ while mental illness and substance use are more frequently associated with homelessness among White individuals.^33^ These differing paths to homelessness may contribute to more adverse health-related circumstances and higher rates of mortality among White PEH. Second, the mortality risk in the general population among Black individuals far surpasses the mortality risk among White individuals.^34^ This increased risk of death in the general Black population, owing to myriad factors, likely contributes to a comparatively less stark mortality disparity observed among Black PEH.

### Limitations

Our study has limitations. Causes of death were based on death certificate data and subject to the accuracy of these records. Deaths that occurred outside of Massachusetts were not captured. Findings are representative of data collected between 2003 and 2018 and should be considered as relevant to policies active during that timeframe. While all individuals in the study cohort accessed homeless-tailored services at least once during the study period, we did not have data regarding the extent of clinical engagement. We lacked information on individual clinical characteristics as well as social and structural determinants of health that may have influenced mortality. In particular, housing status is a dynamic phenomenon that was not longitudinally tracked for individual cohort members. However, the inclusion of individuals who may have regained housing over the study period may have conservatively biased our comparisons with the general population. Additionally, some individuals in the general population who experienced homelessness throughout the life-course were not included in our cohort. Because we evaluated adults who accessed tailored primary care services for PEH in Boston, our findings may not be generalizable to homeless-experienced individuals in other geographic areas or those who do not receive care at a Health Care for the Homeless program. Although the overall large cohort size allowed for stratified analyses, relatively small sample sizes in some strata may have resulted in less-precise mortality estimates for certain demographic subgroups.

## Conclusions

In this large cohort study of PEH, all-cause mortality rates differed by age, gender, and race and ethnicity. Drug overdose mortality was a leading cause of death among all individuals regardless of age, gender, and race and ethnicity. Younger individuals were heavily affected by suicide, while Black and Hispanic/Latinx individuals were heavily affected by homicide and HIV. Policies aimed toward reducing homelessness and interventions tailored to consider the unique needs and preferences of distinct sociodemographic groups of PEH are necessary to address preventable mortality disparities in this population.

## Notes

Supplement 1. **eTable 1.** *International Classification of Diseases* (*ICD*)-*10* Codes for Underlying Cause of Death **eTable 2.** Mortality Rate Differences for the Five Leading Causes of Death Among BHCHP Women Compared to Women in the Urban Northeast US Population, 2003-2018 **eTable 3.** Mortality Rate Differences for the Five Leading Causes of Death Among BHCHP Men Compared to Men in the Urban Northeast US Population, 2003-2018

Supplement 2.
Data Sharing Statement

## References

[1] Henry M, De Sousa T, Roddey C, . The 2020 Annual Homeless Assessment Report (AHAR) to Congress. January 2021. Accessed August 1, 2023. https://www.huduser.gov/portal/sites/default/files/pdf/2020-AHAR-Part-1.pdf

[2] National Alliance to End Homelessness. State of homelessness: 2021 edition. Accessed August 1, 2023. https://endhomelessness.org/homelessness-in-america/homelessness-statistics/state-of-homelessness-2021/

[3] The Aspen Institute. The COVID-19 eviction crisis: an estimated 30-40 million people in America are at risk. August 7, 2020. Accessed August 1, 2023. https://www.aspeninstitute.org/blog- posts/the-covid-19-eviction-crisis-an-estimated-30-40-million-people-in-america-are-at-risk/

[4] Lebrun-Harris LA, Baggett TP, Jenkins DM, . Health status and health care experiences among homeless patients in federally supported health centers: findings from the 2009 patient survey. Health Serv Res. 2013;48(3):992-1017. doi:10.1111/1475-6773.12009 23134588 PMC3681240

[5] Fazel S, Geddes JR, Kushel M. The health of homeless people in high-income countries: descriptive epidemiology, health consequences, and clinical and policy recommendations. Lancet. 2014;384(9953):1529-1540. doi:10.1016/S0140-6736(14)61132-6 25390578 PMC4520328

[6] Baggett TP, Hwang SW, O’Connell JJ, . Mortality among homeless adults in Boston: shifts in causes of death over a 15-year period. JAMA Intern Med. 2013;173(3):189-195. doi:10.1001/jamainternmed.2013.1604 23318302 PMC3713619

[7] Hibbs JR, Benner L, Klugman L, . Mortality in a cohort of homeless adults in Philadelphia. N Engl J Med. 1994;331(5):304-309. doi:10.1056/NEJM199408043310506 8022442

[8] Hwang SW, Orav EJ, O’Connell JJ, Lebow JM, Brennan TA. Causes of death in homeless adults in Boston. Ann Intern Med. 1997;126(8):625-628. doi:10.7326/0003-4819-126-8-199704150-00007 9103130

[9] Barrow SM, Herman DB, Córdova P, Struening EL. Mortality among homeless shelter residents in New York City. Am J Public Health. 1999;89(4):529-534. doi:10.2105/AJPH.89.4.529 10191796 PMC1508869

[10] Kasprow WJ, Rosenheck R. Mortality among homeless and nonhomeless mentally ill veterans. J Nerv Ment Dis. 2000;188(3):141-147. doi:10.1097/00005053-200003000-00003 10749278

[11] Schinka JA, Leventhal KC, Lapcevic WA, Casey R. Mortality and cause of death in younger homeless veterans. Public Health Rep. 2018;133(2):177-181. doi:10.1177/0033354918755709 29420922 PMC5871144

[12] Los Angeles County Department of Public Health, Center for Health Impact Evaluation, Recent Trends In Mortality Rates and Causes of Death Among People Experiencing Homelessness in Los Angeles County. January 2021. Accessed August 1, 2023. http://www.publichealth.lacounty.gov/chie/reports/HomelessMortality2020_CHIEBrief_Final.pdf

[13] Dickins KA, Fine DR, Adams LD, . Mortality trends among adults experiencing homelessness in Boston, Massachusetts From 2003 to 2018. JAMA Intern Med. 2023;183(5):488-490. doi:10.1001/jamainternmed.2022.7011 36912831 PMC10012038

[14] Fine DR, Dickins KA, Adams LD, . Drug overdose mortality among people experiencing homelessness, 2003 to 2018. JAMA Netw Open. 2022;5(1):e2142676. doi:10.1001/jamanetworkopen.2021.42676 34994792 PMC8742197

[15] Hwang SW, Wilkins R, Tjepkema M, O’Campo PJ, Dunn JR. Mortality among residents of shelters, rooming houses, and hotels in Canada: 11 year follow-up study. BMJ. 2009;339:b4036. doi:10.1136/bmj.b4036 19858533 PMC2767481

[16] Roncarati JS, Baggett TP, O’Connell JJ, . Mortality among unsheltered homeless adults in Boston, Massachusetts, 2000-2009. JAMA Intern Med. 2018;178(9):1242-1248. doi:10.1001/jamainternmed.2018.2924 30073282 PMC6142967

[17] O’Connell JJ, Oppenheimer SC, Judge CM, . The Boston Health Care for the Homeless Program: a public health framework. Am J Public Health. 2010;100(8):1400-1408. doi:10.2105/AJPH.2009.173609 20558804 PMC2901289

[18] Boston Health Care for the Homeless Program. About us. Accessed August 1, 2023. https://www.bhchp.org/about-us

[19] Dusetzina SB, Tyree S, Meyer AM, Meyer A, Green L, Carpenter WR. Linking data for health services research: a framework and instructional guide. Agency for Healthcare Research and Quality, report No. 14-EHC033-EF. September 2014. Accessed August 3, 2023. https://www.ncbi.nlm.nih.gov/books/NBK253313/pdf/Bookshelf_NBK253313.pdf 25392892

[20] National Center for Health Statistics. National Death Index User’s Guide; 2013.

[21] Fine DR, Lewis E, Weinstock K, Wright J, Gaeta JM, Baggett TP. Office-based addiction treatment retention and mortality among people experiencing homelessness. JAMA Netw Open. 2021;4(3):e210477. doi:10.1001/jamanetworkopen.2021.0477 33662132 PMC7933994

[22] Baggett TP, Chang Y, Singer DE, . Tobacco-, alcohol-, and drug-attributable deaths and their contribution to mortality disparities in a cohort of homeless adults in Boston. Am J Public Health. 2015;105(6):1189-1197. doi:10.2105/AJPH.2014.302248 25521869 PMC4431083

[23] Centers for Disease Control and Prevention [CDC]. Underlying cause of death 1999-2019. Accessed August 1, 2023. https://wonder.cdc.gov/wonder/help/ucd.html

[24] Heron M. Deaths: leading causes for 2017. Natl Vital Stat Rep. 2019;68(6):1-77. 32501203

[25] US Department of Health and Human Services [HHS], Centers for Disease Control and Prevention [CDC]. CDC Wide-ranging ONline Data for Epidemiologic Research (CDC WONDER). 2021. Accessed August 1, 2023. https://wonder.cdc.gov/

[26] Cameron AC, Trivedi PK. Regression Analysis of Count Data. Cambridge University Press; 1998. doi:10.1017/CBO9780511814365

[27] Spinelli MA, Ponath C, Tieu L, Hurstak EE, Guzman D, Kushel M. Factors associated with substance use in older homeless adults: results from the HOPE HOME study. Subst Abus. 2017;38(1):88-94. doi:10.1080/08897077.2016.1264534 27897965 PMC5472372

[28] Poon G, Holleran L, Chu J, Goldblum P, Bongar B. A qualitative analysis of suicide risk factors, preferred means, and means restriction feasibility within a homeless shelter environment. J Soc Distress Homeless. 2017;26(2):148-156. doi:10.1080/10530789.2017.1363505

[29] Vogel M, Thompson KJ, Messner SF. The enduring influence of cohort characteristics on race-specific homicide rates. Soc Forces. 2020;99(1):1-30. doi:10.1093/sf/soz127

[30] Siddiqi AE, Hu X, Hall HI; Centers for Disease Control and Prevention (CDC). Mortality among Blacks or African Americans with HIV infection—United States, 2008-2012. MMWR Morb Mortal Wkly Rep. 2015;64(4):81-86. 25654607 PMC4584853

[31] Crenshaw K. Demarginalizing the intersection of race and sex: a Black feminist critique of antidiscrimination doctrine, feminist theory and antiracist politics. Univ Chic Leg Forum. 1989;1989:8. August 1, 2023. https://chicagounbound.uchicago.edu/uclf/vol1989/iss1/8

[32] Olivet J, Wilkey C, Richard M, . Racial inequity and homelessness: findings from the SPARC Study. Annals Am Acad Pol. 2021;693(1):82-100. doi:10.1177/0002716221991040

[33] North CS, Smith EM. Comparison of White and non-White homeless men and women. Soc Work. 1994;39(6):639-647. 7992133

[34] Benjamins MR, Silva A, Saiyed NS, De Maio FG. Comparison of all-cause mortality rates and inequities between Black and White populations across the 30 most populous US cities. JAMA Netw Open. 2021;4(1):e2032086. doi:10.1001/jamanetworkopen.2020.32086 33471116 PMC9386890

