# Error in Figure 2

**DOI:** 10.1001/jamanetworkopen.2023.38506

**Published:** 2023-10-03

In the Original Investigation titled “Mortality by Age, Gender, and Race and Ethnicity in People Experiencing Homelessness in Boston, Massachusetts,”^1^ published August 31, 2023, there was an error in the x-axis of Figure 2. The first grouping of data given as “All men” should have been “All women.” This article has been corrected.^1^
