# Supplementary material for: Mortality by Age, Gender, and Race and Ethnicity in People Experiencing Homelessness in Boston, Massachusetts
Source: JAMA Netw Open. 2023 Aug 31;6(8):e2331004. doi: 10.1001/jamanetworkopen.2023.31004 (PMC10472188; doi:10.1001/jamanetworkopen.2023.31004)
Supplement: Supplement 1. — eTable 1. International Classification of Diseases (ICD)-10 Codes for Underlying Cause of Death eTable 2. Mortality Rate Differences for the Five Leading Causes of Death Among BHCHP Women Compared to Women in the Urban Northeast US Population, 2003-2018 eTable 3. Mortality Rate Differences for the Five Leading Causes of Death Among BHCHP Men Compared to Men in the Urban Northeast US Population, 2003-2018 [file jamanetwopen-e2331004-s001.pdf]

Supplementary Online Content

Fine DR, Dickins KA, Adams LD, et al. Mortality by age, gender, race, and ethnicity in people experiencing homelessness. *JAMA Netw Open*. 2023;6(8):e2331004. doi:10.1001/jamanetworkopen.2023.31004

eTable 1. *International Classification of Diseases* (*ICD*)*-10* Codes for Underlying Cause of Death

eTable 2. Mortality Rate Differences for the 5 Leading Causes of Death Among Women in the BHCHP Cohort Compared to Women in the Urban Northeast US Population, 2003-2018

eTable 3. Mortality Rate Differences for the 5 Leading Causes of Death Among Men in the BHCHP Cohort Compared to Men in the Urban Northeast US Population, 2003-2018

This supplementary material has been provided by the authors to give readers additional information about their work.

© 2023 Fine DR et al. *JAMA Network Open.*

eTable1. *International Classification of Diseases* (*ICD*)*-10* Codes for Underlying Cause of Death		
**Underlying Cause of Death Categorization**		**ICD-10**
Natural Causes		
Certain infectious and parasitic diseases (A00-B99)	Sepsis	A40-A41
	Viral hepatitis	B15-B19
	HIV disease	B20-B24
	Other and unspecified infectious and parasitic diseases and their sequelae	A00, A05, A20-A36, A42-A44, A48-A49, A54-A79, A81-A82, A85.0-A85.1, A85.8, A86-B04, B06-B09, B25-B49, B55-B99
Neoplasms (C00-D48)	Malignant neoplasms of lip, oral cavity, pharynx, larynx	C00-C14, C32
	Malignant neoplasm of esophagus	C15
	Malignant neoplasms of colon, rectum and anus	C18-C21
	Malignant neoplasms of liver and intrahepatic bile ducts	C22, D37.6
	Malignant neoplasm of pancreas	C25
	Malignant neoplasms of trachea, bronchus and lung	C33-C34, D38.1
	Malignant neoplasm of breast, cervix uteri, corpus uteri and uterus, ovary	C50, C53-C56
	Malignant neoplasm of prostate	C61
	Malignant neoplasms of lymphoid, hematopoietic and related tissue	C81-C96
	All other and unspecified malignant neoplasms (e.g., bladder, brain, stomach, skin, kidney and renal pelvis)	C17, C23-C24, C26-C31, C37-C41, C44-C49, C51-C52, C57-C60, C62-C63, C66, C68-C69, C73-C80, C97
Diseases of the blood (D50-D89)	Diseases of the blood (anemias, hemorrhagic conditions, disorders involving the immune mechanism)	D50-D89
Endocrine, nutritional and metabolic diseases (E00-E90)	Diabetes mellitus	E10-E14
	All other endocrine, nutritional and metabolic diseases	E00-E32, E34.0-E34.2, E34.4-E34.9, E65-E83, E85, E88
Mental and behavioral disorders	Dementia and delirium	F01-F05, G30
	Psychoactive substance use disorder	F10-F19

© 2023 Fine DR et al. *JAMA Network Open.*

(F00-F99)	Alcohol use disorder	F10
	Other substance use disorders	F11-F19
	Other and unspecified mental and behavioral disorders	F06-F07, F09, F20-F48, F50-F99
Diseases of the nervous system (G00-G99)	Anoxic brain injury	G93.1
	Other diseases of nervous system	G04, G06-G11, G12.1-G12.9, G20-G72, G81-G92, G93.0, G93.2-G93.9, G95-G98
Diseases of the circulatory system (I00-I99)	Major cardiovascular diseases	I00-I78
	Cerebrovascular diseases	I60-I69
	Other disorders of circulatory system	I80-I99
Diseases of the respiratory system (J00-J99)	Influenza and pneumonia	J09-J18
	Chronic lower respiratory diseases (COPD, chronic bronchitis and asthma)	J40-J47
	Other and unspecified diseases of respiratory system	J00-J06, J30-J39, J67, J70-J98
Diseases of the digestive system (K00-K93)	Liver disease	K70-K77
	All other and unspecified diseases of digestive system	K00-K14, K20-K31, K35-K38, K40-K46, K50-K52, K55-K67, K80-K87, K90-K93
Diseases of the skin and subcutaneous tissue (L00-L99)	Diseases of the skin and subcutaneous tissue	L00-L99
Diseases of the musculoskeletal system and connective tissue (M00-M99)	Diseases of the musculoskeletal system and connective tissue	M00-M99
Diseases of the genitourinary system (N00-N99)	Renal failure and other disorders of kidney	N17-N19, N25, N27
	Other and unspecified diseases of genitourinary system	N00-N15, N20-N23, N26, N28-N95
Pregnancy, childbirth and the puerperium (O00-O99)	Pregnancy, childbirth and the puerperium	O00-O99
Congenital malformations, deformations and chromosomal abnormalities (Q00-Q99)	Congenital malformations, deformations and chromosomal abnormalities	Q00-Q99

© 2023 Fine DR et al. *JAMA Network Open.*

Symptoms, signs and abnormal clinical and laboratory findings, not elsewhere classified (R00-R99)	Symptoms, signs and abnormal clinical and laboratory findings, not elsewhere classified	R00-R99
External Causes		
Accidents (V01-X59, Y10-Y34)	Transport accidents	V01-V99, Y85
	Non-transport accidents (non-poisoning)	W00-X59, Y86
	Drug overdose	X40-X44, Y10-Y14
	Alcohol poisoning	X45, Y15
Intentional self-harm (X60-X84)	Suicide	U03, X60-X84, Y87.0
Assault (X85-Y09)	Homicide	U01-U02, X85-Y09, Y87.1
Legal interventions and operations of war (Y35-Y36)	Legal interventions and operations of war	Y35, Y89.0
Complications of medical and surgical care (Y40-Y84)	Complications of medical and surgical care	Y40-Y84, Y88
Adapted from: Heron M. Deaths: Leading causes for 2017. National Vital Statistics Reports; vol 68 no 6. Hyattsville, MD: National Center for Health Statistics. 2019.		

© 2023 Fine DR et al. *JAMA Network Open.*

eTable 2. Mortality Rate Differences for the 5 Leading Causes of Death Among Women in the BHCHP Cohort Compared to Women in the Urban Northeast US Population, 2003-2018								
	Entire BHCHP Cohort		Black, Non-Hispanic/Latinx		Hispanic/Latinx		White, Non-Hispanic/Latinx	
Age strata	COD	Rate Difference (95% CI)	COD	Rate Difference (95% CI)	COD	Rate Difference (95% CI)	COD	Rate Difference (95% CI)
18-34	Drug overdose	182.7 (151.1-214.2)	Drug overdose	24.7 (1.4-48.0)	Drug overdose	81.2 (39.4-122.9)	Drug overdose	444.4 (357.2-531.6)
	PSUD	34.9 (21.2-48.5)	Diabetes	12.6 (-3.8-29.1)	PSUD	15.5 (-2.6-33.5)	PSUD	68.8 (34.8-102.8)
	Suicide	17.1 (6.8-27.5)	Homicide	6.5 (-10.0-22.9)	NA	NA	Suicide	39.5 (12.6-66.4)
	Ill-defined	10.7 (2.7-18.7)	CVD	7.1 (-9.4-23.5)	NA	NA	Ill-defined	33.1 (9.0-57.2)
	Other accidents[a]	1.5 (-5.6-8.6)	PSUD	13.8 (-2.7-30.2)	NA	NA	Other accidents[a]	9.5 (-9.5-28.5)
35-49	Drug overdose	285.9 (244.1-327.7)	Drug overdose	118.2 (66.2-170.2)	Drug overdose	136.0 (74.1-198.0)	Drug overdose	566.6 (469.4-663.7)
	CVD	50.3 (29.6-71.1)	CVD	17.6 (-19.2-54.4)	Liver disease	58.1 (17.5-98.6)	PSUD	131.2 (84.7-177.6)
	PSUD	68.1 (47.7-88.4)	Cancer	-16.8 (-50.6-17.1)	CVD	40.4 (2.2-78.6)	CVD	87.5 (46.4-128.6)
	Cancer	8.4 (-10.2-26.9)	HIV	14.6 (-16.0-45.2)	PSUD	38.8 (5.7-71.9)	Cancer	27.5 (-7.4-62.3)
	Liver disease	38.5 (22.5-54.5)	PSUD[b]	26.8 (1.8-51.7)	HIV	21.9 (-8.4-52.1)	Liver disease	56.4 (24.6-88.2)
50-64	Cancer	64.9 (13.6-116.2)	Cancer	60.4 (-38.0-158.8)	CVD	66.2 (-34.4-166.8)	Drug overdose	349.9 (259.5-440.4)
	Drug overdose	226.2 (178.9-273.6)	CVD	84.0 (-5.6-173.6)	Drug overdose	100.7 (13.6-187.8)	Cancer	80.0 (-2.9-162.9)
	CVD	125.5 (78.3-172.6)	Drug overdose	119.9 (57.4-182.3)	Liver disease	94.7 (7.6-181.8)	Liver disease	213.0 (141.2-284.8)
	Liver disease	129.3 (92.5-166.0)	HIV	46.9 (1.5-92.2)	Cancer	-49.4 (-129.0-30.1)	CVD	132.8 (61.9-203.6)
	PSUD	69.1 (42.9-95.3)	Liver disease	48.7 (5.7-91.7)	PSUD	69.3 (-1.8-140.4)	Lower resp.	85.4 (36.6-134.1)
65-79	Cancer	-12.0 (-181.6-157.6)	Cancer	93.6 (-272.8-460.0)	CVD	-180.8 (-439.4-77.9)	Drug overdose	88.4 (-14.1-191.0)

© 2023 Fine DR et al. *JAMA Network Open.*

	CVD	-56.4 (-204.3-91.5)	CVD	-196.9 (-496.1-102.3)	NA	NA	CVD	108.3 (-156.5-373.2)
	Lower resp.	82.3 (-10.6-175.2)	Cerebrovascular disease	61.5 (-111.3-234.2)	NA	NA	Lower resp.	231.8 (26.7-437.0)
	Liver disease	75.2 (7.3-143.0)	Sepsis	74.5 (-75.0-224.1)	NA	NA	Liver disease	127.3 (-5.1-259.7)
	Cerebrovascular disease	2.3 (-61.2-65.8)	NA	NA	NA	NA	Viral hepatitis[c]	87.6 (-15.0-190.2)

[a] Other accidents ICD-10: W00-X59, Y16-Y36, Y86.
[b] Tie in number of deaths; cause of death with largest rate difference displayed in table. Other cause = lower respiratory disease, rate difference: 25.3 (0.3-50.3)
[c] Tie in number of deaths; cause of death with largest rate difference displayed in table. Other causes = Cancer, rate difference: 68.9 (-227.3-365.0); Diabetes, rate difference: 41.1 (-61.5-143.6); Other digestive diseases, rate difference: 48.6 (-54.0-151.2).
Abbreviation legend: “BHCHP”=Boston Health Care for the Homeless Program; “CVD”=cardiovascular disease; “HIV”= human immunodeficiency virus; “Lower resp.”=chronic lower respiratory disease; “PSUD”=psychoactive substance use disorder; “NA”=not available
“PSUD” includes ICD-10 codes for mental and behavioral disorders due to psychoactive substance use (codes F10-19) and is generally intended for deaths related to a chronic pattern or sequelae of substance use rather than acute poisoning which is documented as “drug overdose” (codes X40-X44, Y10-Y14)

© 2023 Fine DR et al. *JAMA Network Open.*

eTable 3. Mortality Rate Differences for the 5 Leading Causes of Death Among Men in the BHCHP Cohort Compared to Men in the Urban Northeast US Population, 2003-2018

	Entire BHCHP Cohort		Black, Non-Hispanic/Latinx		Hispanic/Latinx		White, Non-Hispanic/Latinx	
Age strata	COD	Rate Difference (95% CI)	COD	Rate Difference (95% CI)	COD	Rate Difference (95% CI)	COD	Rate Difference (95% CI)
18-34	Drug overdose	466.4 (410.4, 522.4)	Drug overdose	103.1 (45.9-160.3)	Drug overdose	204.4 (124.4-284.3)	Drug overdose	763.8 (656.7-871.0)
	Suicide	36.8 (19.1-54.5)	Homicide	-22.5 (-73.8-28.7)	PSUD	43.3 (6.9-79.6)	Suicide	68.6 (34.2-103.0)
	PSUD	47.3 (29.6-64.9)	CVD	23.0 (-10.0-56.0)	Ill-defined	19.7 (-6.0-45.4)	PSUD	69.9 (37.9-101.9)
	Homicide	4.1 (-11.0-19.1)	Suicide	9.7 (-15.9-35.3)	Homicide	1.0 (-24.7-26.7)	Ill-defined	52.4 (23.9-80.8)
	Ill-defined	28.2 (13.9-42.5)	Cancer	13.8 (-11.8-39.4)	NA	NA	Transport accident	16.0 (-3.4-35.4)
35-49	Drug overdose	398.1 (361.4-434.8)	Drug overdose	108.8 (68.2-149.4)	Drug overdose	280.4 (212.6-348.2)	Drug overdose	656.3 (584.0-728.6)
	CVD	59.0 (40.0-78.0)	CVD	-4.8 (-38.7-29.1)	CVD	24.8 (-5.7-55.4)	CVD	114.0 (79.0-148.9)
	PSUD	73.3 (57.4-89.2)	Cancer	-8.2 (-32.2-15.7)	PSUD	42.4 (14.7-70.1)	PSUD	140.6 (107.3-173.8)
	Cancer	26.3 (11.7-40.8)	HIV	-10.3 (-32.1-11.5)	HIV	23.8 (-3.0-50.5)	Liver disease	93.3 (65.2-121.4)
	Liver disease	46.8 (33.3-60.2)	Homicide	-6.6 (-25.9-12.7)	Cancer	5.9 (-17.5-29.3)	Cancer	52.2 (25.9-78.4)
50-64	CVD	176.8 (139.3-214.3)	Cancer	22.6 (-39.7-84.9)	Drug overdose	246.0 (162.3-329.7)	CVD	372.2 (307.8-436.7)
	Cancer	144.0 (107.9-180.1)	CVD	-82.3 (-140.5--24.1)	Cancer	56.7 (-22.1-135.5)	Cancer	247.0 (188.3-305.6)
	Drug overdose	288.6 (256.3-320.8)	Drug overdose	130.5 (86.4-174.6)	Liver disease	122.7 (57.6-187.8)	Drug overdose	412.3 (357.5-467.1)
	PSUD	159.0 (135.2-182.7)	PSUD	68.9 (37.8-100.1)	CVD	-34.9 (-98.8-29.1)	PSUD	260.3 (217.2-303.4)

© 2023 Fine DR et al. *JAMA Network Open.*

	Liver disease	129.6 (106.4-152.8)	Liver disease	43.3 (14.0-72.7)	HIV	50.8 (3.0-98.6)	Liver disease	201.2 (160.9-241.4)
65-79	Cancer	185.8 (56.0-315.6)	Cancer	288.6 (3.3-573.9)	Cancer	-55.8 (-343.4-231.9)	CVD	366.1 (174.5-557.7)
	CVD	174.3 (44.5-304.1)	CVD	-192.3 (-439.0-54.5)	CVD	-250.0 (-511.2-11.1)	Cancer	166.3 (-12.8-345.5)
	Lower resp.	103.8 (43.6-164.1)	Diabetes	79.7 (-42.7-202.0)	Lower resp.	174.1 (-10.3-358.6)	Lower resp.	134.5 (43.3-225.6)
	Diabetes	59.1 (8.8-109.4)	Lower resp.	12.8 (-79.0-104.5)	PSUD	128.2 (-11.2-267.6)	Drug overdose	149.9 (82.0-217.8)
	Drug overdose	115.1 (71.0-159.2)	Renal failure[a]	22.9 (-58.0-103.8)	Cerebrovascular disease	53.3 (-86.1-192.8)	Other resp. diseases[b]	84.3 (19.9-148.8)

[a]Tie in number of deaths; cause of death with largest rate difference displayed in table. Other causes = Cerebrovascular disease, rate difference: -46.8 (-127.9-34.2)

[b]Other resp. diseases ICD-10: J00-J06, J20-J22, J30-J39, J60-J70, J80-J84, J85-J86, J90-J94, J96-J98.

Abbreviation legend: “BHCHP”=Boston Health Care for the Homeless Program; “CVD”=cardiovascular disease; “HIV”= human immunodeficiency virus; “Lower resp”=chronic lower respiratory disease; “PSUD”=psychoactive substance use disorder; “NA”=not available

“PSUD” includes ICD-10 codes for mental and behavioral disorders due to psychoactive substance use (codes F10-19) and is generally intended for deaths related to a chronic pattern or sequelae of substance use rather than acute poisoning which is documented as “drug overdose” (codes X40-X44, Y10-Y14)

© 2023 Fine DR et al. *JAMA Network Open.*
