# Supplementary material for: Mortality by Age, Gender, and Race and Ethnicity in People Experiencing Homelessness in Boston, Massachusetts
Source: JAMA Netw Open. 2023 Aug 31;6(8):e2331004. doi: 10.1001/jamanetworkopen.2023.31004 (PMC10472188; doi:10.1001/jamanetworkopen.2023.31004)
Supplement: Supplement 2. — Data Sharing Statement [file jamanetwopen-e2331004-s002.pdf]

Data Sharing Statement

Fine. Mortality by Age, Gender, and Race and Ethnicity in People Experiencing Homelessness in Boston, Massachusetts. *JAMA Netw Open*. Published August 31, 2023. doi:10.1001/jamanetworkopen.2023.31004

Data
Data available: Yes
Data types: Deidentified participant data
How to access data: The data that support the findings of this study are available from the corresponding author, KD (Kirsten_Dickins@rush.edu), upon reasonable request.
When available: With publication

Supporting Documents
Document types: None

Additional Information
Who can access the data: Deidentified data will be made available to researchers whose proposed use of the data has been approved by the study team.
Types of analyses: Any type of analysis will be considered by the study team.
Mechanisms of data availability: The data will be made available without investigator support, after approval of a proposal, and with a signed data access agreement.
